# Change in exhaled nitric oxide during peanut challenge is related to severity of reaction

**DOI:** 10.1186/s13223-020-00464-8

**Published:** 2020-07-21

**Authors:** Elizabeth Percival, Rani Bhatia, Kahn Preece, Mark McEvoy, Adam Collison, Joerg Mattes

**Affiliations:** 1 GrowUpWell Priority Research Centre, Hunter Medical Research Institute, University of Newcastle, Lot 1 Kookaburra Circuit, New Lambton Heights, Newcastle, NSW 2305 Australia; 2 Department of Paediatric Medicine, John Hunter Children’s Hospital, Newcastle, NSW Australia; 3 Department of Paediatric Allergy & Immunology, John Hunter Children’s Hospital, Newcastle, NSW Australia; 4 School of Medicine and Public Health, University of Newcastle, Newcastle, NSW Australia; 5 Department of Paediatric Respiratory & Sleep Medicine, John Hunter Children’s Hospital, Newcastle, NSW Australia

**Keywords:** Peanut, Allergy, Anaphylaxis, FeNO, Fraction exhaled nitric oxide, Skin prick test, Ara h2 sIgE, Peanut sIgE

## Abstract

**Background:**

Peanut allergy affects 3% of Australian children and has a higher risk of anaphylaxis than most food allergies. Predicting who is likely to develop anaphylaxis is still an inexact science. The fraction of exhaled nitric oxide (FeNO) shows promise as a biomarker involved in peanut allergy, as nitric oxide plays a role in inhibiting mast cell degranulation which is relevant in anaphylaxis, where mast cell degranulation plays a mediator role. The aim of this study was to assess the change in FeNO in children during peanut challenge.

**Methods:**

Thirty-six children aged from 5 to 17 years were recruited for open-labelled peanut challenge. Participants had skin prick test to peanut performed, and serum collected for Ara h2 specific IgE and peanut specific IgE. FeNO was measured by portable device (NIOX VERO) prior to and throughout the peanut challenge.

**Results:**

When grouped according to reaction type at peanut challenge (anaphylaxis, clinical allergy not anaphylaxis and tolerant), there were significant differences in the mean change in FeNO measurement between the anaphylaxis group and the clinical allergy, not anaphylaxis group (p = 0.005), and between the anaphylaxis group and tolerant group (p < 0.0001).

**Conclusions:**

FeNO decreased more significantly in those who subsequently developed anaphylaxis than in those with clinical allergy, not anaphylaxis or negative peanut challenge (tolerance). As a bedside test that can be used in children, it has potential for further research into mechanisms of anaphylaxis in food allergy and potentially assists in predicting an imminent anaphylactic reaction in some patients.

*Trial registration* ClinicalTrials.gov: PEAnut Anaphylaxis Predictors (PEAAP) NCT02424136.

## Background

Peanut allergy is one of the most common food allergies, reported to affect up to 3% of Australian children in a 2011 population-based study [1]. While food allergy related fatalities are rare, peanut remains the most common cause and reactions remain unpredictable [2]. Unlike other food allergies such as dairy and egg which often resolve by school age [3–8], peanut allergy tends to persist with tolerance developing in approximately 20% of children [9], but the mechanisms involved are not yet apparent [10].

Diagnosing peanut allergy requires a convincing history of an IgE-mediated allergic reaction after ingestion of peanut and demonstration of IgE sensitisation to peanut through skin prick testing (SPT) or serum peanut specific IgE (sIgE) [11]. IgE sensitisation can be present in the absence of clinical allergy, [12] thereby making the history of reaction essential to diagnosis. The gold standard for diagnosis of food allergy continues to be a double-blind placebo controlled oral food challenge [13, 14]. Food challenges have an inherent risk of severe allergic reaction, are time consuming for patients, their family and health care professionals, and are financially costly to health care services in providing an appropriately equipped environment and staff for the duration of the challenge. To limit time and financial cost, it is routine clinical practice to perform open label food challenges after assessing the likelihood of allergic reaction with peanut ingestion [1, 15, 16]. Evidence suggests that the likelihood of an allergic reaction can be estimated by the size of the peanut SPT wheal and by the level of serum peanut sIgE [17, 18]. Unfortunately the increasing size of the wheal or level of serum sIgE does not always correlate with the severity of the reaction at food challenge [12, 19].

Component resolved diagnostics, which use sIgE levels to specific components of the peanut protein (particularly Ara h2) have shown promise for improving the capacity to predict risk of allergic reaction to peanut but have limitations in predicting likelihood of reaction in clinical practice [11, 20, 21].

Asthma is a known risk factor for severe allergic reactions [22]. Fraction of exhaled nitric oxide (FeNO) is a non-invasive measure of eosinophilic airway inflammation clinically utilised in the diagnosis and monitoring of asthma [23], but is also associated with IgE sensitisation [24, 25]. FeNO has been shown to be elevated in children with peanut allergy who have “outgrown” their asthma [26]. Our research group has previously shown that FeNO may also improve the ability to predict allergic reaction during peanut challenge [15, 27]. The mechanism for this is not clear, but asthma did not appear to be necessarily involved as the area under the curve for FeNO with all children (0.89) was similar to the area under the curve when asthmatic children were excluded (0.90) [27].

Nitric oxide (NO) is produced by multiple cell types in the respiratory tract, including epithelial, inflammatory and vascular endothelial cells [28]. It is generated by nitric oxide synthases (NOS) oxidising l-arginine and depending on the isoform (neural, inducible or endothelial), the effect can be proinflammatory (inducible NOS or NOS2) or physiological (neural NOS or NOS1, and endothelial NOS or NOS3) [28]. Nitric oxide is also involved in the inhibition of mast cell degranulation and mediator release, which is relevant to anaphylaxis where mast cell degranulation is a key mediator [29, 30]. FeNO has been demonstrated to be lower after positive food challenge [31] and after anaphylaxis [32] but there is no published data on what happens to FeNO during food challenge. The association of the decrease in FeNO following positive food challenge is hypothesised to be related to the early phase of inflammation, but the exact mechanism is not yet known [31].

The primary aim of this study was to assess the change in FeNO during peanut challenge in children with a clinical need for peanut challenge (such as to clarify where the history is lacking or ambiguous, or to assess for the development of tolerance).

## Methods

### Study population

Thirty-nine children aged from 5 to 17 years were recruited for open-labelled peanut challenge at a tertiary referral paediatric allergy centre in Newcastle, Australia. Children were offered participation in the study following assessment by their allergy specialist or paediatrician as suitable for a graded supervised challenge. Their food challenge had been scheduled to (1) confirm a peanut allergy diagnosis in those whose history of reaction was ambiguous, (2) assess for the possibility of acquired tolerance, or (3) test for clinical reactivity in children who had not consumed peanut but were sensitised. Participants were excluded from the study if their SPT to whole peanut extract was > 10 mm due to the high likelihood of clinical reaction. The cohort included 28 patients with a history of an IgE-mediated reaction to peanut, not within the last 12 months.

### Ethics and consent

The Hunter New England Human Research Ethics Committee and University of Newcastle Human Research Ethics Committee approved the study. Informed written consent was obtained from all parents or guardians prior to entry into the study, and from children as appropriate for their age. The study was registered with ClinicalTrials.gov as PEAnut Anaphylaxis Predictors (PEAAP) NCT02424136.

### Pre-challenge assessment

Participants underwent a pre-challenge assessment on either the morning of the peanut challenge or the day before the challenge. The assessment included a clinical questionnaire focusing on their personal and family history of atopy, by use of modified version of a previously validated parental questionnaire [33, 34]. Allergic rhinitis was assessed using paediatric validated allergic rhinitis and its impact on asthma (ARIA) criteria [35] where symptoms are classified according to symptom frequency (intermittent or persistent) and severity (mild or moderate-severe). A score is then allocated 1—intermittent mild, 2—intermittent moderate/severe, 3—persistent mild or 4—persistent moderate/severe. Eczema history was assessed based upon previous medical diagnosis and the current treatment required, other than emollients. Visible eczema was assessed using the validated SCORAD Index [36]. Asthma history was assessed by use of the modified version of a previously validated parental questionnaire [33, 34]. It assessed doctor diagnosed asthma, the presence of wheezing ever and the number of wheezing episodes in the previous 12 months. Further focus was given to wheeze causing sleep disturbance, limiting daily activities, and associated with exercise; as well as dry nocturnal cough not related to respiratory infection, seeking medical attention for cough or wheeze, and the use of a reliever, preventer or oral steroids in the previous 12 months. Current diagnosis of asthma referred to those patients who needed use of reliever, preventer or oral steroids for asthma in the previous 12 months.

Participants then underwent FeNO measurement, pre- and post-bronchodilator spirometry, peanut SPT, and blood collection for serum peanut and Ara h2 sIgE. FeNO was measured according to the American Thoracic Society and European Respiratory Society (ATS/ERS) guidelines [37] by a portable electrochemical analyser, NIOX VERO (Circassia AB, Uppsala, Sweden). FeNO measurement with NIOX VERO required participants to empty their lungs then inhale deeply through the filter to total lung capacity. They then exhaled into the device for 10 s at an exhalation pressure of 10–20 cm H_2_O to maintain a fixed flow rate of 50 ± 5 mL/s, with the assistance of visual and audible feedback incorporated into the device. FeNO measurement with NIOX VERO was repeated 10 min after each dose of peanut in the food challenge until they completed the challenge or developed signs of allergic reaction and stopped the challenge. The value in parts per billion was recorded.

Spirometry was performed on the MasterScreen PFT (Vyaire Medical, Mettawa, Illinois USA) according to ATS/ERS guidelines [37] for the standardisation of spirometry.

Peanut SPT was performed on the volar surface of the participant’s forearm, using standard whole peanut extract reagent, 1:10 w/v (Stallergenes Greer, London, United Kingdom). A positive test was a wheal size 3 mm greater than the negative control. The wheal size was determined by averaging maximal perpendicular wheal diameter 15 min after applying the lancet (Stallergenes Greer, London, United Kingdom). Positive control was with histamine base, 6 mg/mL (Stallergenes Greer, London, United Kingdom) and with a wheal ≥ 3 mm indicating a valid test [38]. Negative control was glycerol saline.

Blood was collected for serum testing and analysed using ImmunoCAP 250 system (Phadia AB, Uppsala, Sweden) for peanut sIgE and Ara h2 sIgE.

### Food challenge

The open label peanut challenge was conducted according to Australasian Society of Clinical Immunology and Allergy (ASCIA) food challenge protocol for peanut [39]. This involves an incremental increase in dose ingested every 20 min throughout the challenge. Initial dose is a smear of peanut butter inside lip, then 0.625 g, with doubling of increment size every 20 min up to a maximum dose of 5 g. A medical doctor supervised all challenges. Challenge results were classified positive or negative according to predefined criteria from the PRACTALL consensus report [14], where a positive outcome at challenge was defined by the presence of signs (or persisting symptoms) of clinical allergy. A negative outcome at challenge was defined by the absence of signs during the challenge and 2 h after ingestion of all peanut doses. A follow up phone call was made the next day to ensure no late reactions occurred. These participants were declared tolerant. Positive challenge results were further categorised as anaphylaxis (defined according to ASCIA guidelines [40]) or clinical allergy not anaphylaxis (CANA) (any other positive challenge result that was not anaphylaxis).

### Statistics

STATA 15.1 (StataCorp, College Station, Texas, USA) and Prism 8 for macOS (GraphPad Software, La Jolla California, USA) were used for statistical analysis and graphical presentation. Participant clinical features are presented as medians with minimum and maximum values for continuous variables and frequency with percentages for categorical variables. Differences between groups (defined by their results at peanut challenge) were tested with Mann–Whitney two-tailed test for continuous variables that were not normally distributed and Fisher’s exact test for categorical variables. For calculating differences in normally distributed continuous variables between groups, the unpaired t-test was used.

Receiver operator characteristic (ROC) curves were produced in STATA 15.1 and used to assess the ability of clinical investigations prior to challenge and FeNO at each time point prior to and during the food challenge, to predict a positive challenge and anaphylaxis to peanuts. The area under the curve (AUC) is a summary measure of the combined sensitivity and specificity for all possible cut points.

To estimate the ability of combined clinical investigations (peanut SPT, Ara h2 sIgE, FeNO) in predicting a positive challenge or anaphylaxis at challenge, a logistic regression model was fitted with ROC curves generated from the results with STATA 15.1.

Summary data graphs were produced in Prism 8 for macOS to show the mean change (and mean percentage change) in FeNO for each group (defined by results at peanut challenge) from the prechallenge assessment to each timepoint post incremental ingestion of peanut through the peanut challenge. Difference between groups was tested with 2-way ANOVA. Differences between groups at individual timepoints were tested with Mann–Whitney two-tailed test.

As there were no previous studies measuring FeNO during food challenge, there was a lack of data from which to predict differences between groups at food challenge and hence no power calculation was performed prior to the study.

## Results

### Peanut challenges

Thirty-nine children were recruited to the study, and 38 challenges were performed in 37 children (Table 1). Two children were unable to undergo peanut challenge: one child was unwell on the day of the challenge and was unable to be rescheduled during the study timeframe; a second child’s peanut SPT was > 10 mm on the day of challenge, so not challenged as per study exclusion criteria. A third child participated in the study early and returned 3 years later for a repeat challenge, only data from their second challenge was included for analysis, as their FeNO testing was incomplete at their first challenge. A fourth child’s challenge result was equivocal, so their data was excluded from the analysis (more information on this case can be found in Additional file 1). This left data from 36 challenges (22 males, 14 females) available for analysis. Twenty-one of the challenges were positive (clinical allergy) and when grouped according to severity, 9 had anaphylaxis, while 12 had CANA.

**Table 1:** Participant clinical features

Clinical feature	Entire cohort (n = 36)
Age (years)	
Median (min, max)	10.2 (5.1, 17.1)
Gender (%)	
Males	22 (61)
Previous peanut ingestion (%)	
Total	28 (78)
Previous adrenaline usage (%)	
Total	1 (3)
Other food allergy (%)	
Total	7 (19)
Household smokers (%)	
Total	7 (19)
Allergic rhinitis (%)	
Total	26 (72)
AR severity for those with AR-max = 4^a^	
Median (min, max)	2 (1, 4)
Eczema ever (%)	
Total	29 (81)
Eczema active treatment (%)	
Total	8 (22)
SCORAD for those with visible eczema	
Median (min, max)	14.7 (6.0, 36.0)
Asthma ever (%)	
Total	15 (42)
Current preventer (%)	
Total	8 (22)
Current reliever (%)	
Total	10 (28)
Exercise related wheeze or dry nocturnal cough (%)^b^	
Total	16 (44)
No allergy in challenge (%)	
Total	15 (42)
CANA in challenge (%)	
Total	12 (33)
Anaphylaxis in challenge (%)	
Total	9 (25)
Peanut SPT (mm)	
Median (min, max)	5.8 (0, 10.0)
Peanut sIgE (kU/L)	
Median (min, max)	1.35 (0.01, 92.00)
Ara h2 sIgE (kU/L)	
Median (min, max)	0.85 (0.00, 70.30)
FeNO (NIOX VERO^c^) (ppb)	
Median (min, max)	23 (5, 97)
Percent predicted FEV1^d^	
Median (min, max)	102 (72, 124)
Percent predicted FVC^d^	
Median (min, max)	100 (70, 128)
Percent predicted FEV1/FVC^d^	
Median (min, max)	100 (82, 114)

*AR* allergic rhinitis, *SCORAD* SCORing Atopic Dermatitis *SPT* skin prick test, *sIgE* specific immunoglobulin E, *FeNO* fraction exhaled nitric oxide, *p.p.b* Parts per billion. *FEV1* forced expiratory volume in 1 s, *FVC* forced vital capacity

^a^ AR severity: 1 = intermittent mild, 2 = intermittent mod-severe, 3 = persistent mild, 4 = persistent mod-severe

^b^ Exercise related wheeze or dry nocturnal cough, not related to respiratory infection in the previous 12 months

^c^ Only 33 patients were able to have FeNO measured via NIOX VERO

^d^ Only 21 patients were able to have spirometry performed

### Data availability

Data for peanut SPT, peanut sIgE, and Ara h2 sIgE were available for all 36 participants. Due to young age, poor technical ability and limited time available prior to food challenge, 33 participants had data available for FeNO measured via NIOX VERO and 21 participants had spirometry performed. One participant who had a negative challenge missed a FeNO measurement following the 1.25 g dose.

### Participant clinical features

Participant clinical features are outlined in Table 1. The median age was 10.2 years (range 5.1–17.1 years). Twenty-eight (78%) children had eaten peanut before, but only 1 had previously been given adrenaline for an allergic reaction to peanut. Comorbid atopic conditions were common: 26 (72%) had a history of allergic rhinitis, 29 (81%) had history of eczema, with 8 (22%) actively treating eczema. Fifteen (42%) had a history of doctor diagnosed asthma, with 10 (28%) using a reliever and 8 (22%) using a preventer in the last 12 months. There were an additional 5 (14%) children who reported symptoms of exercise related wheeze or dry nocturnal cough not related to respiratory infection, despite not having doctor diagnosed asthma (not shown on table). Of these 5 children, 2 developed CANA, while 3 were tolerant at challenge.

When grouped according to result at food challenge (Table 2), there were significant differences between the groups for peanut SPT wheal size (tolerant 3.5 mm compared to clinical allergy 7.0 mm, p = 0.0001), peanut sIgE (tolerant 0.50 kU/L compared to clinical allergy 3.20 kU/L, p = 0.005) and Ara h2 sIgE (tolerant 0.13 kU/L compared to clinical allergy 1.60 kU/L, p = 0.0002). All other clinical features were not significantly different.

**Table 2:** Participant clinical features by outcome of food challenge

	Tolerant (n = 15)	Clinical allergy (n = 21)	p-value
Age (years)			
Median (min, max)	10.2 (7.5, 17.2)	11.4 (5.1, 16.5)	0.9054
Gender (%)			
Males	12 (80)	10 (48)	0.0833
Previous peanut ingestion (%)			
Total	12 (80)	16 (76)	> 0.9999
Previous adrenaline usage (%)			
Total	1 (7)	0 (0)	0.4167
Other food allergy (%)			
Total	2 (13)	5 (24)	0.6738
Household smokers (%)			
Total	3 (20)	4 (19)	> 0.9999
Allergic rhinitis (%)			
Total	11 (73)	15 (71)	> 0.9999
AR severity for those with AR-max = 4^a^			
Median (min, max)	1 (1, 4)	2 (1, 4)	0.6860
Eczema ever (%)			
Total	11 (73)	18 (86)	0.4178
Eczema active treatment (%)			
Total	3 (20)	5 (24)	> 0.9999
SCORAD for those with visible eczema			
Median (min, max)	15.3 (10.7, 36.0)	14.7 (6.0, 32.5)	0.6429
Asthma ever (%)			
Total	8 (53)	7 (33)	0.3104
Current preventer (%)			
Total	4 (27)	4 (19)	0.6940
Current reliever (%)			
Total	6 (40)	4 (19)	0.2600
Exercise related wheeze or dry nocturnal cough (%)^b^			
Total	8 (53)	8 (38)	0.4996
Peanut SPT (mm)			
Median (min, max)	3.5 (0.0, 10.0)	7.0 (4.0, 9.0)	*0.0001*
Peanut sIgE (kU/L)			
Median (min, max)	0.50 (0.01, 26.80)	3.20 (0.10, 92.00)	*0.0054*
Ara h2 sIgE (kU/L)			
Median (min, max)	0.13 (0.00, 1.00)	1.60 (0.08, 70.30)	*0.0002*
FeNO (NIOX VERO^c^) (p.p.b)			
Median (min, max)	22 (5, 49)	29 (5, 97)	0.2662
Percent predicted FEV1^d^			
Median (min, max)	100 (86, 121)	102 (72, 124)	0.7910
Percent predicted FVC^d^			
Median (min, max)	104 (88, 128)	100 (70, 119)	0.4147
Percent predicted FEV1/FVC^d^			
Median (min, max)	95 (82, 114)	102 (93, 111)	0.0692

*AR* allergic rhinitis, *SCORAD* SCORing Atopic Dermatitis *SPT* skin prick test, *sIgE* specific Immunoglobulin E, *FeNO* fraction exhaled nitric oxide, *FEV1* forced expiratory volume in 1 s, *FVC* forced vital capacity

^a^ AR severity: 1 = intermittent mild, 2 = intermittent mod-severe, 3 = persistent mild, 4 = persistent mod-severe

^b^ Exercise related wheeze or dry nocturnal cough, not related to respiratory infection in the previous 12 months

^c^ Only 33 patients were able to have FeNO measured via NIOX VERO (14 tolerant, 19 clinical allergy)

^d^ Only 21 patients were able to have spirometry performed (6 tolerant, 15 clinical allergy)

Italic text indicates significant p value (less than 0.05)

After grouping participants based upon severity of reaction at food challenge (tolerant, CANA or anaphylaxis), peanut SPT and Ara h2 sIgE were significantly lower in those who were tolerant than those with CANA (3.5 mm vs 7.0 mm, p = 0.001, and 0.13 kU/L vs 1.25 kU/L, p = 0.002 respectively), and in those who were tolerant than those with anaphylaxis (3.5 mm vs 7.0 mm, p = 0.003 and 0.13 kU/L vs 1.80 kU/L p = 0.003 respectively), but not between those with CANA and those with anaphylaxis (7.0 mm vs 7.0 mm, p = 0.63 and 1.25 kU/L vs 1.80 kU/L p = 0.92 respectively). The peanut sIgE was significantly lower only between those who were tolerant and those with anaphylaxis (0.50 kU/L vs 4.8 kU/L p = 0.005) (Table 3).

**Table 3:** Participant clinical features by severity of reaction at food challenge

	Tolerant (n = 15)	CANA (n = 12)	p value (tolerant v CANA)	Anaphylaxis (n = 9)	p value (tolerant v anaphylaxis)	p value (CANA v anaphylaxis)
Age (years)						
Median (min, max)	10.2 (7.5, 17.2)	11.5 (6.9, 16.5)	0.6054	9.2 (5.1, 14.8)	0.3863	0.3918
Gender (%)						
Males	12 (80)	6 (50)	0.1266	4 (44)	0.0994	> 0.9999
Previous peanut ingestion (%)						
Total	12 (80)	10 (83)	> 0.9999	6 (67)	0.6349	0.6108
Previous adrenaline usage (%)						
Total	1 (7)	0 (0)	> 0.9999	0 (0)	> 0.9999	> 0.9999
Other food allergy (%)						
Total	2 (13)	2 (17)	> 0.9999	3 (33)	0.3256	0.6108
Household smokers (%)						
Total	3 (20)	2 (17)	> 0.9999	2 (22)	> 0.9999	> 0.9999
Allergic rhinitis (%)						
Total	11 (73)	8 (67)	> 0.9999	7 (78)	> 0.9999	0.6591
AR severity for those with AR-max = 4^a^						
Median (min, max)	1 (1, 4)	2 (1, 4)	0.6599	2 (1, 4)	0.9340	0.7436
Eczema ever (%)						
Total	11 (73)	10 (83)	0.6618	8 (89)	0.6146	> 0.9999
Eczema active treatment (%)						
Total	3 (20)	2 (17)	> 0.9999	3 (33)	0.6349	0.6108
SCORAD for those with visible eczema						
Median (min, max)	15.3 (10.7, 36.0)	9.2 (6.0, 19.1)	0.4000	16.2 (13.1, 32.5)	0.9143	0.4000
Asthma ever (%)						
Total	8 (53)	4 (33)	0.4408	3 (33)	0.4225	>0.9999
Current preventer (%)						
Total	4 (27)	4 (33)	> 0.9999	0 (0)	0.2589	0.1038
Current reliever (%)						
Total	6 (40)	4 (33)	> 0.9999	0 (0)	0.0519	0.1038
Exercise related wheeze or dry nocturnal cough (%)^b^						
Total	8 (53)	6 (50)	> 0.9999	2 (22)	0.2099	0.3666
Peanut SPT (mm)						
Median (min, max)	3.5 (0.0, 10.0)	7.0 (4.0, 9.0)	*0.0011*	7.0 (5.0, 9.0)	*0.0029*	0.6344
Peanut sIgE (kU/L)						
Median (min, max)	0.50 (0.01, 26.80)	1.47 (0.10. 92.00)	0.0541	4.80 (0.37, 80.60)	*0.0051*	0.6640
Ara h2 sIgE (kU/L)						
Median (min, max)	0.13 (0.00, 1.00)	1.25 (0.10, 70.30)	*0.0017*	1.80 (0.08, 64.50)	*0.0025*	0.9170
FeNO (NIOX^c^) (p.p.b)						
Median (min, max)	22 (5, 49)	15 (5, 71)	0.8822	38 (9, 97)	0.0591	0.2133
Percent predicted FEV1^d^						
Median (min, max)	100 (86, 121)	99 (78, 123)	0.6354	108 (72, 124)	0.9307	0.5941
Percent predicted FVC^d^						
Median (min, max)	104 (88, 128)	99 (76, 112)	0.2100	114 (70, 119)	0.9307	0.3556
Percent predicted FEV1/FVC^d^						
Median (min, max)	95 (82, 114)	105 (95, 111)	0.0765	102 (93, 103)	0.2273	0.2394

Italic text indicates significant p value (less than 0.05)

*AR* allergic rhinitis, *CANA* clinical allergy not anaphylaxis, SCORAD SCORing Atopic Dermatitis *SPT* skin prick test, *sIgE* serum specific immunoglobulin E, *FeNO* fraction exhaled nitric oxide

^a^ AR severity: 1 = intermittent mild, 2 = intermittent mod-severe, 3 = persistent mild, 4 = persistent mod-severe

^b^ Exercise related wheeze or dry nocturnal cough, not related to respiratory infection in the previous 12 months

^c^ Only 33 patients were able to have FeNO measured via NIOX (14 tolerant, 11 CANA, 8 anaphylaxis)

^d^ Only 21 patients were able to have spirometry performed (6 tolerant, 10 CANA, 5 anaphylaxis)

Interestingly, none of the participants who developed anaphylaxis had a current diagnosis of asthma (Table 3). Three of the participants who developed anaphylaxis had a previous diagnosis of asthma that had since resolved clinically, with no use of reliever or preventer in the previous 12 months (Table 3). While 2 of the 9 children with anaphylaxis reported a dry nocturnal cough not related to respiratory infection over the previous 12 months (and had previously been diagnosed with asthma by a medical doctor), only 1 of the 9 with anaphylaxis had a history of needing admission to hospital for asthma (not intensive care) and none had used a reliever or preventer medication in the last 12 months. (This last child was 1 of the 2 patients with anaphylaxis who reported dry nocturnal cough not related to respiratory infection). In the normally distributed FeNO, despite there being no current asthmatics in the anaphylaxis group, FeNO measured prechallenge was significantly higher (p = 0.02, by parametric analysis, not shown on table) in the anaphylaxis group (38 ppb) than in the tolerant group (22 ppb).

### Mean change in FeNO throughout peanut challenge

**Fig. 1:**
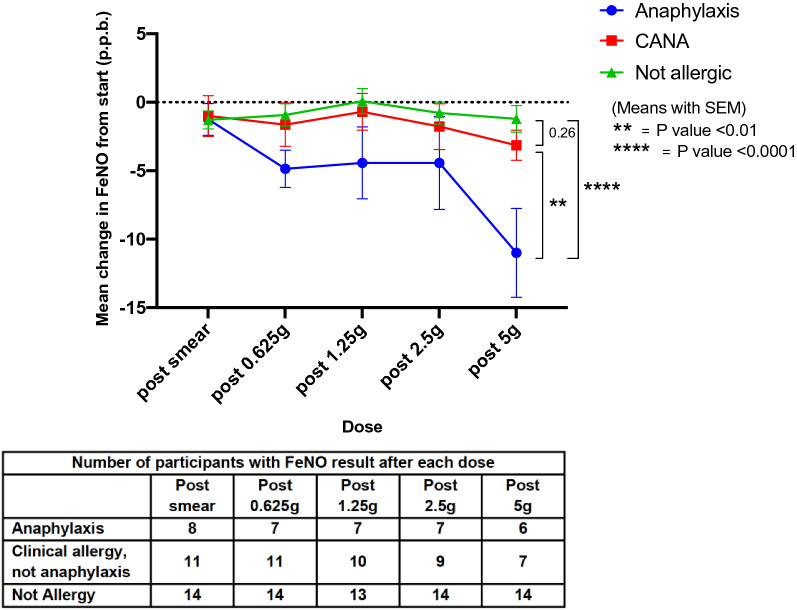
Mean change in FeNO (p.p.b) at each increment. Grouped according to reaction type at challenge

When grouped according to reaction type at peanut challenge (tolerant, CANA and anaphylaxis), the mean change in FeNO in the anaphylaxis group was significantly lower than both the CANA group (p = 0.005) and tolerant group (p < 0.0001) but there was no significant difference between CANA and tolerant group (p = 0.26) (Fig. 1).

When comparing the median differences between groups at individual timepoints in the challenge, FeNO decreased significantly more in the anaphylaxis group than tolerant group after 0.625 g increment (minus 4 ppb compared with minus 1 ppb, p = 0.006) and after 5 g increment (minus 9 ppb compared with minus 1 ppb, p = 0.003). The other timepoints (post smear, post 1.25 g and post 2.5 g) were not significantly different. FeNO also decreased significantly more in the anaphylaxis group compared to the CANA group after 5 g increment (minus 9 ppb compared with minus 3 ppb, p = 0.009). The other timepoints (post smear, post 0.625 g, post 1.25 g and post 2.5 g) were not significantly different.

After stratifying participants only by current asthma diagnosis, there was no significant difference in mean change in FeNO throughout the challenge between groups, although the non-asthmatic participants were trending towards a lower mean value (p = 0.059) (Additional file 1: Figure S1). Similarly, after stratifying participants only by doctor diagnosed asthma ever, there was no significant difference in mean change in FeNO throughout the challenge between groups, although the non-asthmatic participants were also trending towards a lower mean value at the end of the challenge (p = 0.1054) (Additional file 1: Figure S2).

There was no significant difference in mean FeNO change in the anaphylaxis group when divided into those with lower respiratory tract signs (cough, wheeze or hypoxia) and those without (p > 0.99).

### Mean percentage change in FeNO throughout peanut challenge

**Fig. 2:**
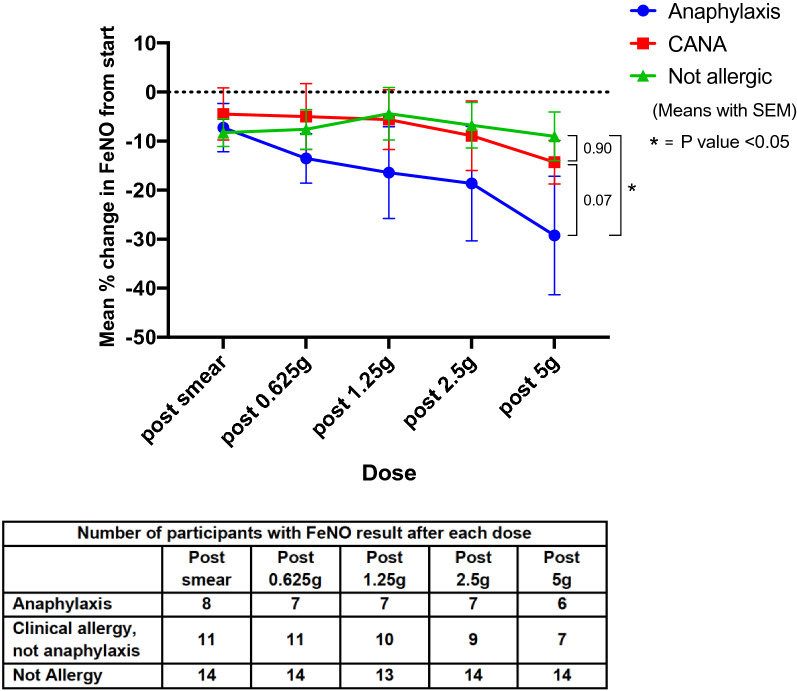
Mean percentage change in FeNO (p.p.b) at each increment. Grouped according to reaction type at challenge

When grouped according to reaction type at peanut challenge (tolerant, CANA and anaphylaxis), there was a significant difference in the mean percentage change in FeNO measurement between the anaphylaxis group and tolerant group (p = 0.01) (Fig. 2). There was no significant difference in the mean percentage change in FeNO measurement between the anaphylaxis group and the CANA group (p = 0.07), or between the CANA group and the tolerant group (p = 0.90). The median differences in percentage change between groups at individual timepoints in the challenge were not significantly different.

### Accuracy of FeNO prior to and throughout challenge for predicting outcome

The AUC of FeNO prior to challenge for predicting clinical allergy was 0.62 (95% CI 0.42–0.82) which compared with the AUC for post smear of peanut butter of 0.63 (95% CI 0.43–0.83), post 0.625 g peanut butter of 0.63 (95% CI 0.43–0.83), post 1.25 g peanut butter of 0.66 (95% CI 0.46–0.87), post 2.5 g peanut butter of 0.62 (95% CI 0.41–0.83), and post 5 g peanut butter of 0.59 (95% CI 0.35–0.82) (Additional file 1: Figure S3).

The AUC of FeNO prior to challenge for predicting anaphylaxis was 0.72 (95% CI 0.48–0.96) which compared with the AUC for post smear of peanut butter of 0.72 (95% CI 0.49–0.96), post 0.625 g peanut butter of 0.73 (95% CI 0.50–0.97), post 1.25 g peanut butter of 0.71 (95% CI 0.45–0.96), post 2.5 g peanut butter of 0.69 (95% CI 0.41–0.98), and post 5 g peanut butter of 0.67 (95% CI 0.33–1.00) (Additional file 1: Figure S4).

### Accuracy of tests prior to challenge for predicting outcome

The AUC for SPT, sIgE, Ara h2 and FeNO predicting clinical allergy ranged between 0.62 and 0.86 (Additional file 1: Figure S5).

**Fig. 3:**
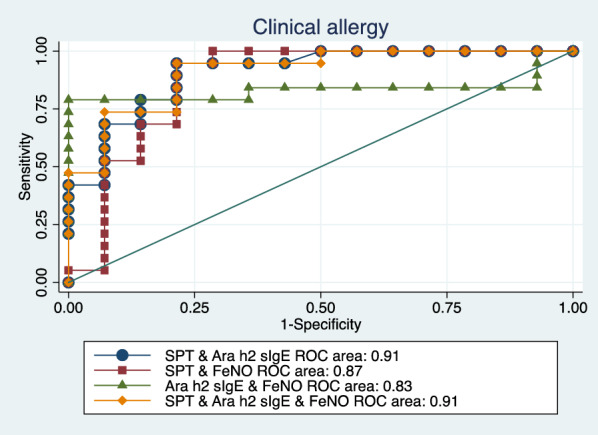
Comparison of combined diagnostic methods by logistic regression analysis, then ROC curve generation for clinical allergy

ROC curves after logistic regression for combined clinical investigations for predicting clinical allergy are show in Fig. 3. When combined with Peanut SPT, both Ara h2 sIgE and FeNO individually (AUC 0.91 and 0.87 respectively) and in combination (AUC 0.91) improved the AUC for predicting clinical allergy.

The AUC for SPT, sIgE, Ara h2 and FeNO predicting anaphylaxis ranged between 0.67 and 0.72 (Additional file 1: Figure S6).

**Fig. 4:**
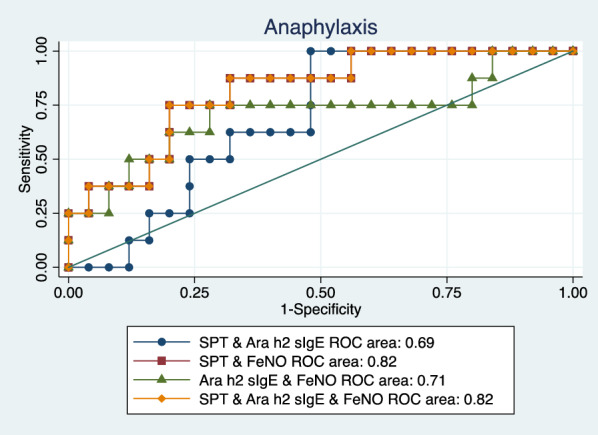
Comparison of combined diagnostic methods by logistic regression analysis, then ROC curve generation for anaphylaxis

ROC curves after logistic regression for combined clinical investigations for predicting anaphylaxis are show in Fig. 4. When combined with Peanut SPT, both Ara h2 sIgE and FeNO individually (AUC 0.69 and 0.82 respectively) and in combination (AUC 0.82) improved the AUC for predicting anaphylaxis.

## Discussion

To the best of our knowledge this is the first study to measure FeNO progressively during peanut challenge in children and to use a portable device for measurement prior to peanut challenge. This study has shown that FeNO decreased significantly more during peanut challenge in children who developed anaphylaxis than those with CANA or tolerance. Our research group has previously shown that FeNO measured prechallenge can improve prediction of positive peanut challenge [15]. Similarly, Benhamou et al. [31] demonstrated that FeNO was higher when measured prechallenge in those with a positive food challenge than in those with a negative challenge. They also demonstrated that FeNO was significantly lower 60 and 90 min after positive food challenge than in those with a negative food challenge. However in their study, there was no association with the FeNO level prechallenge (or change post challenge) and the severity of reaction at food challenge, which could be related to the participants in their study having less severe reactions (up to grade 3 using Sampson’s classification) [41] than in this study (up to grade 4).

Nitric oxide is produced physiologically in low levels by epithelial and inflammatory cells in the airways following the oxidisation of l-arginine by constitutively expressed isoforms of NOS [32, 42, 43]. In contrast, a different isoform—inducible NOS, is expressed with inflammation and drives endothelial cell production of NO in large amounts [32, 43, 44]. This causes increased smooth muscle cyclic guanosine monophosphate, which leads to vascular smooth muscle relaxation [23, 42, 43]. While this mechanism of vasodilatation has been confirmed in septic shock and has been presumed to be involved in anaphylactic shock [44], a mouse study [45] has identified that endothelial NOS was the critical mediator of anaphylaxis in the absence of inducible NOS.

Interestingly, although the prechallenge FeNO concentration was higher in the anaphylaxis group than the tolerant group, it decreased relatively more through the challenge in the anaphylaxis group than in the tolerant group, bringing the two groups means closer together at the completion of the challenge. This demonstrated that the change in FeNO is the differentiating factor between groups rather than the whole FeNO concentration at the completion of the challenge. A similar finding was shown by Benhamou et al. [31], where a significant difference between FeNO in those patients with positive and negative challenge was only evident prechallenge. The median FeNO of each group became closer post challenge meaning that it decreased relatively more in those with a positive challenge.

Importantly, the decrease in FeNO observed in the anaphylaxis group though the challenge was significant regardless of whether they had cough, wheeze or hypoxia during anaphylaxis suggesting independence from bronchoconstriction. This is in contrast to a study in Japan [32] that measured FeNO within 24 h of anaphylaxis of any cause who found higher FeNO levels in those with lower respiratory tract signs or symptoms than without during anaphylaxis.

This study adds support to the utility of FeNO measurement (in this study with a portable device) in combination with routine clinical investigations such as peanut SPT and Ara h2 sIgE prior to peanut challenge for predicting positive challenge. In previous cohorts [15, 27] prechallenge FeNO measurement aligned with positive result at food challenge (but not severity of reaction). However, in this cohort it appears more closely aligned with anaphylaxis, both when used individually or in combination with peanut SPT and Ara h2 sIgE. There are several possible explanations including different devices with different analyser techniques (chemiluminescence device compared with electrochemical sensor device), potentially different population clinical characteristics and/or the relatively small sample size of both studies demonstrating an underpowered result.

While elevated FeNO has more commonly been associated with eosinophilic airways inflammation in asthma, in this study there were no current asthmatics (by history) in the anaphylaxis group, yet they had higher FeNO measurements prechallenge than the tolerant group, suggesting that exhaled nitric oxide is related to food induced anaphylaxis even in the absence of asthma which is considered a strong risk factor for anaphylaxis [2, 46, 47].

The lack of current asthmatics in this group with anaphylaxis is not typical of the reported association between asthma and food allergies, [48] or severe or fatal anaphylaxis [49–51]. It may suggest that this study population is not representative of the general population, alternatively, it may be that there were children with unrecognised asthma symptoms as suggested by the 2 children in the anaphylaxis group with reported dry nocturnal cough not related to respiratory infection over the previous 12 months.

This study also reaffirms the validity of Ara h2 sIgE in predicting a positive result at peanut challenge, with lesser capacity to predict severity of reaction. This correlates with systematic reviews [21, 52] on component resolved diagnostics in peanut allergy, although the capacity to predict severity of reaction is not consistent across studies.

FeNO is a quick non-invasive test. In a portable device with visual display it is easy to administer even to children. FeNO concentration has been used clinically in the diagnosis and monitoring of asthma but is also associated with IgE sensitisation [24, 25, 53]. The role exhaled nitric oxide plays in food allergy is not yet clear. Its use in oral food challenges has the potential to lead to earlier warning of impending anaphylaxis, although larger repeat studies would be necessary to determine criteria for abandoning a food challenge based upon change in FeNO measurement. As there is variability in dosing and timing of dosing in oral food challenges across clinical practice [14], using a standardised approach, such as advised in the PRACTALL consensus report [14] would be necessary. A limitation for the use of FeNO in very young children is the developmental skills necessary to perform the test, by modulating expiratory flow rate in response to visual or auditory cues. This restricts the use of the test in a large group of children undergoing oral food challenge.

Nitric oxide has many roles including regulating pulmonary vascular tone, airway smooth muscle tone, and inhibition of mast cell degranulation and mediator release [29, 30]. As mast cell degranulation is a key mediator of anaphylaxis, NO is a protective factor important in inhibiting anaphylaxis and the decrease in FeNO seen in the participants who developed anaphylaxis may be a warning that they are approaching anaphylaxis. Despite thorough literature review, we have not been able to determine the mechanism for this association. We are confident that the participants’ technique using the FeNO analyser did not deteriorate through the challenge as the device required a fixed flow rate of 50 ± 5 mL/s to provide a valid measurement. Pathophysiologically, it is known that bronchial hyperreactivity (by methacholine challenge) increases following a food challenge, even in those with a normal FEV1 immediately prior to food challenge [54]. Furthermore, the hyperreactivity was evident without lower respiratory tract symptoms in some patients, suggesting that airway inflammation can exist asymptomatically in some patients. We hypothesise that the decrease in FeNO may be related to changes in the diffusion capacity of nitric oxide, possibly due to oedema of the airway epithelial cells in the early phase of inflammation prior to anaphylaxis. Further research is needed on this topic to clarify the mechanism.

The lack of FeNO measurements right up to the onset of anaphylaxis and during anaphylaxis leaves a gap in understanding what happens to FeNO during anaphylaxis. Other studies have measured FeNO after food challenge [31] or after anaphylaxis [32] with decreased FeNO after positive food challenge and elevated FeNO in those with respiratory symptoms in anaphylaxis respectively. Interestingly, in the patients from the latter study [32] with severe anaphylaxis, FeNO appeared lower within 24 h of anaphylaxis than 1 month later when they were said to be clinically stable. This is congruent with our observations. Regarding measurement of FeNO during allergic reaction in our study, as the participants were children undertaking a clinically indicated food challenge, the decision to prioritise acute management of allergic reaction over measurement of FeNO was made prior to commencing the study.

This study has focussed only on children who had a clinical indication for peanut challenge, i.e. were sensitised to peanut, but may be peanut tolerant with ingestion. Children with high likelihood of reaction (peanut SPT > 10 mm) were excluded from the study. Therefore, the results are not representative of all children with peanut allergy or those with other non-peanut food allergies. The decision to investigate peanut allergy was related to the higher rates of anaphylaxis in peanut allergy than most other food allergies [55].

The use of open labelled peanut challenge rather than double blind placebo controlled peanut challenge is a limitation, however to limit time and financial cost, it is routine clinical practice to perform open label food challenges after assessing the likelihood of allergic reaction with peanut ingestion [1, 16]. To limit the possibility of false positive results, predefined objective clinical criteria [14] were used for classifying outcomes of the challenge.

The lack of a power calculation may also be a limitation for this study, however, as there were no previous studies measuring FeNO during food challenge, there was a lack of data from which to predict differences between groups at food challenge. Despite this, we have been able to demonstrate significant differences in the mean change in FeNO between the anaphylaxis group and the other groups. This study therefore provides the preliminary data for determining power calculations for future studies exploring this association.

## Conclusion

In conclusion, this is the first study to measure FeNO during food challenge and demonstrated that FeNO decreased more significantly in those who subsequently developed anaphylaxis than in those with CANA or negative peanut challenge. As FeNO measurement is a quick bedside test that can be used in children, it lends itself to further research on a diagnostic role in food challenge outcomes. It may also help elucidate the underlying mechanisms in peanut anaphylaxis, potentially assisting in predicting an imminent anaphylactic reaction in patients and improving the safety of oral food challenges.

## Supplementary information

**Additional file 1: Figure S1.** Asthma (current) v non-asthmatic FeNO change each increment. **Figure S2.** Asthma ever v nonasthmatic FeNO change each increment. **Figure S3.** ROC graph for FENO before and during challenge to predict clinical allergy. **Figure S4.** ROC graph for FeNO before and during challenge to predict anaphylaxis. **Figure S5.** ROC graph for prechallenge investigations to predict clinical allergy. **Figure S6.** ROC curves for prechallenge investigations to predict anaphylaxis.

## Data Availability

The data that support the findings of this study are available on request from the corresponding author. The data are not publicly available due to privacy or ethical restrictions.

## Abbreviations

AR: Allergic rhinitis
ARIA: Allergic rhinitis and its impact on asthma
ASCIA: Australasian Society of Clinical Immunology and Allergy
ATS/ERS: American Thoracic Society and European Respiratory Society
AUC: Area under the curve
CANA: Clinical allergy not anaphylaxis
FeNO: Fraction of exhaled nitric oxide
FEV1: Forced expiratory volume in 1 s
FVC: Forced vital capacity
NO: Nitric oxide
NOS: Nitric oxide synthases
p.p.b: Parts per billion
PEAAP: PEAnut Anaphylaxis Predictors
ROC: Receiver operator characteristic
SCORAD: SCORing Atopic Dermatitis
sIgE: Specific IgE
SPT: Skin prick testing
