# Supplementary material for: Change in exhaled nitric oxide during peanut challenge is related to severity of reaction
Source: Allergy Asthma Clin Immunol. 2020 Jul 21;16:64. doi: 10.1186/s13223-020-00464-8 (PMC7386245; doi:10.1186/s13223-020-00464-8)
Supplement: Supplementary file 1 — Additional file 1: Figure S1. Asthma (current) v non-asthmatic FeNO change each increment. Figure S2. Asthma ever v nonasthmatic FeNO change each increment. Figure S3. ROC graph for FENO before and during challenge to predict clinical allergy. Figure S4. ROC graph for FeNO before and during challenge to predict anaphylaxis. Figure S5. ROC graph for prechallenge investigations to predict clinical allergy. Figure S6. ROC curves for prechallenge investigations to predict anaphylaxis. [file 13223_2020_464_MOESM1_ESM.docx]

# Additional file

## Details on equivocal reaction

A 6 year old female with peanut SPT 8 mm, peanut sIgE 2.77 kU/L, Ara h2 sIgE 3.5 kU/L, FeNO 28ppb developed isolated sneezing and rhinorrhea a little more than 2 hours post last dose of peanut. The symptoms resolved without treatment within 20 minutes that day but reoccurred within 1 hour for every subsequent ingestion of peanut on the following days at home. A repeat peanut challenge was performed 2 months later outside of the study where she developed abdominal pain, perioral urticaria and sneezing following the 2.5g dose of peanut.

Figure S1: Asthma (current) v non-asthmatic FeNO change each increment

Figure S2: Asthma ever v nonasthmatic FeNO change each increment

Figure S3: ROC graph for FENO before and during challenge to predict clinical allergy

Figure S4: ROC graph for FeNO before and during challenge to predict anaphylaxis

Figure S5: ROC graph for prechallenge investigations to predict clinical allergy

Figure S6: ROC curves for prechallenge investigations to predict anaphylaxis
